# Overcoming Data Bottlenecks in Genomic Pathogen Surveillance

**DOI:** 10.1093/cid/ciab785

**Published:** 2021-11-25

**Authors:** Ayorinde O Afolayan, Johan Fabian Bernal, June M Gayeta, Melissa L Masim, Varun Shamanna, Monica Abrudan, Khalil Abudahab, Silvia Argimón, Celia C Carlos, Sonia Sia, Kadahalli L Ravikumar, Iruka N Okeke, Pilar Donado-Godoy, David M Aanensen, Anthony Underwood, NIHR Global Health Research Unit on Genomic Surveillance of Antimicrobial Resistance, Harry Harste, Mihir Kekre, Dawn Muddyman, Ben Taylor, Nicole Wheeler, Sophia David, Alejandra Arevalo, Maria Fernanda Valencia, Erik C D Osma Castro, Geetha Nagaraj, Vandana Govindan, Akshata Prabhu, D Sravani, M R Shincy, Steffimole Rose, Kundur N Ravishankar, Anderson O Oaikhena, Jolaade J Ajiboye, Erkison Ewomazino Odih, Marietta L Lagrada, Polle Krystle V Macaranas, Agnettah M Olorosa, Elmer M Herrera, Ali Molloy, John Stelling, Carolin Vegvari

**Affiliations:** 1 Department of Pharmaceutical Microbiology, Faculty of Pharmacy, University of Ibadan, Oyo State, Nigeria; 2 Colombian Integrated Program for Antimicrobial Resistance Surveillance, Centro de Investigatión Tibaitatá, Corporación Colombiana de Investigación Agropecuaria, Tibaitatá, Mosquera, Cundinamarca, Colombia; 3 Antimicrobial Resistance Surveillance Reference Laboratory, Research Institute for Tropical Medicine, Muntinlupa, Philippines; 4 Central Research Laboratory, Kempegowda Institute of Medical Sciences, Bengaluru, India; 5 Centre for Genomic Pathogen Surveillance, Wellcome Genome Campus, Hinxton, Cambridge, United Kingdom; 6 The NIHR Global Health Research Unit for the Genomic Surveillance of Antimicrobial Resistance

**Keywords:** bioinformatics, metadata, whole genome sequencing, WGS, antimicrobial resistance

## Abstract

Performing whole genome sequencing (WGS) for the surveillance of antimicrobial resistance offers the ability to determine not only the antimicrobials to which rates of resistance are increasing, but also the evolutionary mechanisms and transmission routes responsible for the increase at local, national, and global scales. To derive WGS-based outputs, a series of processes are required, beginning with sample and metadata collection, followed by nucleic acid extraction, library preparation, sequencing, and analysis. Throughout this pathway there are many data-related operations required (informatics) combined with more biologically focused procedures (bioinformatics). For a laboratory aiming to implement pathogen genomics, the informatics and bioinformatics activities can be a barrier to starting on the journey; for a laboratory that has already started, these activities may become overwhelming. Here we describe these data bottlenecks and how they have been addressed in laboratories in India, Colombia, Nigeria, and the Philippines, as part of the National Institute for Health Research Global Health Research Unit on Genomic Surveillance of Antimicrobial Resistance. The approaches taken include the use of reproducible data parsing pipelines and genome sequence analysis workflows, using technologies such as Data-flo, the Nextflow workflow manager, and containerization of software dependencies. By overcoming barriers to WGS implementation in countries where genome sampling for some species may be underrepresented, a body of evidence can be built to determine the concordance of antimicrobial sensitivity testing and genome-derived resistance, and novel high-risk clones and unknown mechanisms of resistance can be discovered.

The utility of whole genome sequencing (WGS) for public health purposes has been proposed and implemented in a few countries, mostly to supplement existing methodologies with the expectation that it will become more widely used in the future [1]. The application of bacterial WGS specifically for the purposes of antimicrobial resistance (AMR) surveillance has also been the subject of scientific review [2, 3]. On one hand, these reviews are careful to highlight how WGS is not currently able to completely replace phenotypic antimicrobial sensitivity testing (AST), due to factors such as cost and the lack of concordance between the genotypic-derived and phenotypic results for some bug-drug combinations. For some species, such as *Salmonella* spp, *Staphylococcus aureus*, and *Mycobacterium tuberculosis*, the concordance is very good, but for others, such as *Pseudomonas aeruginosa* or *Acinetobacter baumannii*, the concordance is lower due to an incomplete understanding of mechanisms responsible for resistance [4, 5]. However, while a complete switch from phenotypic to WGS-based AMR surveillance is not likely in the near future, performing WGS of targeted pathogen samples has many benefits. WGS can enhance surveillance by providing information about the determinants responsible for resistance, the “vehicles” that carry them, such as plasmids and other mobile genetic elements, and the clonal lineages within which they are found. This enables the study of the emergence and expansion of AMR with a One Health approach, by facilitating the investigation of possible transfer of AMR between different reservoirs, such as humans, animals, and the environment. Because of this, many public health organizations are enthusiastic to adopt WGS in a targeted way to complement existing phenotypic-based surveillance, as exemplified in the Philippines [6]. Balancing the enthusiasm for adoption are the challenges in the implementation. These include challenges related to laboratory aspects of the process, such as cost and turnaround, and are covered in more detail in [4, 7, 8]. The processes required to transform raw sequence data into human-interpretable results are often grouped under the umbrella term “bioinformatics.” Bioinformatics has its own set of challenges, which may also be barriers to introduction. Several manuscripts detail these, including a review of the steps required for the implementation of bacterial WGS, a recent report by the World Health Organization (WHO) on WGS for AMR surveillance, and others [2–4, 9]. These include availability of computing resources to run analytical pipelines; the wide range of software and associated catalogues of AMR determinants available to determine their presence, often confounded by lack of comprehensive benchmarking; standardization of bioinformatics pipelines used to produce the predictions based on WGS so that results are reproducible, including the use of standard operating procedures; requirements for quality assessment throughout the process from the raw sequence data through to the final interpretation; and trained personnel who are able to run and interpret the analyses.

For any institute starting on the journey toward the implementation of genome-based surveillance, these factors can become bottlenecks that impede adoption. In this manuscript we will describe the barriers that may prevent implementation of the bioinformatics required for WGS-based AMR prediction. Through examples derived from the implementation of WGS within the National Institute for Health Research Global Health Research Unit on Genomic Surveillance of Antimicrobial Resistance (GHRU), we illustrate possible avenues to overcome them. Crucially, and in addition to implementation, the final step in the pathway to WGS adoption is the interpretation of the bioinformatics outputs. This gap is primarily filled by hands-on training to analyze and interpret “real” data produced by bioinformatics processes [10].

## BIOINFORMATICS IMPLEMENTATION CHALLENGES

**Figure 1.**
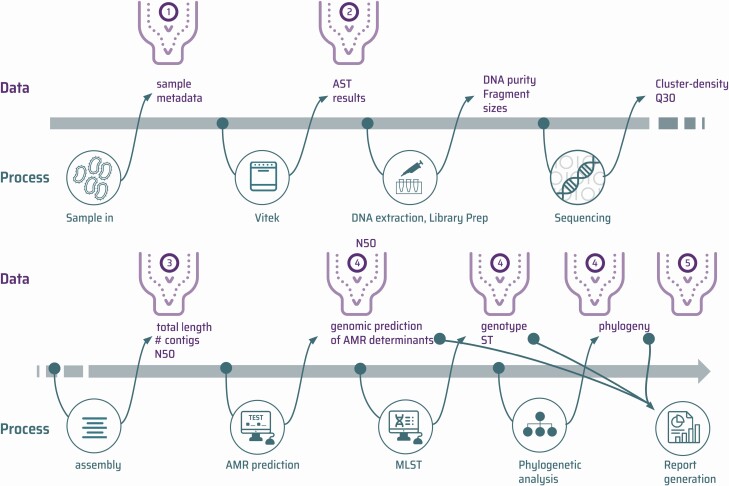
An overview of 1 potential pathway from sample to phenotypic and genomic outputs. The bottleneck icons represent some of the steps in the process that can cause particular implementation challenges. ① Sample metadata cleaning and validation. ② Conversion of antimicrobial sensitivity testing minimum inhibitory concentration data into standardized formats for downstream processing and interpretation. ③ Quantitative quality assessment of raw reads and assemblies. ④ Processing raw reads to detect the presence or absence of genetic loci, genes, specific nonsynonymous mutations, and variants. ⑤ Aggregating results to produce human readable reports. Abbreviations: AMR, antimicrobial resistance; AST, antimicrobial sensitivity testing; MLST, multilocus sequence typing; ST, sequence type.

Laboratory and bioinformatics processes generate various data types when performing pathogen genomics for AMR surveillance as illustrated by an example workflow (Figure 1).

### Data Collection and Integration

Metadata describing samples are critical to contextualize genomics outputs by describing epidemiological and clinical background of samples, allowing association between sample features and genomic markers. Data may be collected at a central site such as a public health laboratory, or by local sample collection sites such as hospitals, and sent to the center performing WGS. In the latter case, data may be stored in a variety of file formats, each structured in different ways. Once AST has been performed, results require merging with sample metadata. Further integration of data will be required later in the process to join these data to the genomic outputs. These data parsing steps can be performed manually but are person-hour expensive and subject to human error. In order to make these processes efficient and minimize turnaround time, automation is crucial.

### Sequence Analysis

Once raw WGS data have been generated, sequence reads need to be processed to derive human interpretable results. This process has often been the hardest to implement, requiring specialist training (see next section), computing infrastructure, and software. Important considerations include using well-defined pipelines so that the outputs are reproducible, and utilizing a solution that is scalable so the hands-on time taken to generate results is, in general, independent of the numbers of samples processed.

### Training

The specialist knowledge required to process WGS data, and understand the databases underpinning interpretation and the principles required when working with big data, is not often a component of the education of many healthcare professionals. If the WGS analysis software has a familiar web-based interface, such as those found on the Galaxy, Centre for Genomic Epidemiology, and Pathogenwatch web applications, less training is required with a focus being on interpretation of the data rather than performing the analysis [11–14]. However, this limits users to the analyses available on the web applications and does not prepare those wanting to perform more in-depth bioinformatics analyses. This requires training in running Linux command-line tools and interpreting the outputs.

## IMPLEMENTATION SOLUTIONS

**Figure 2.**
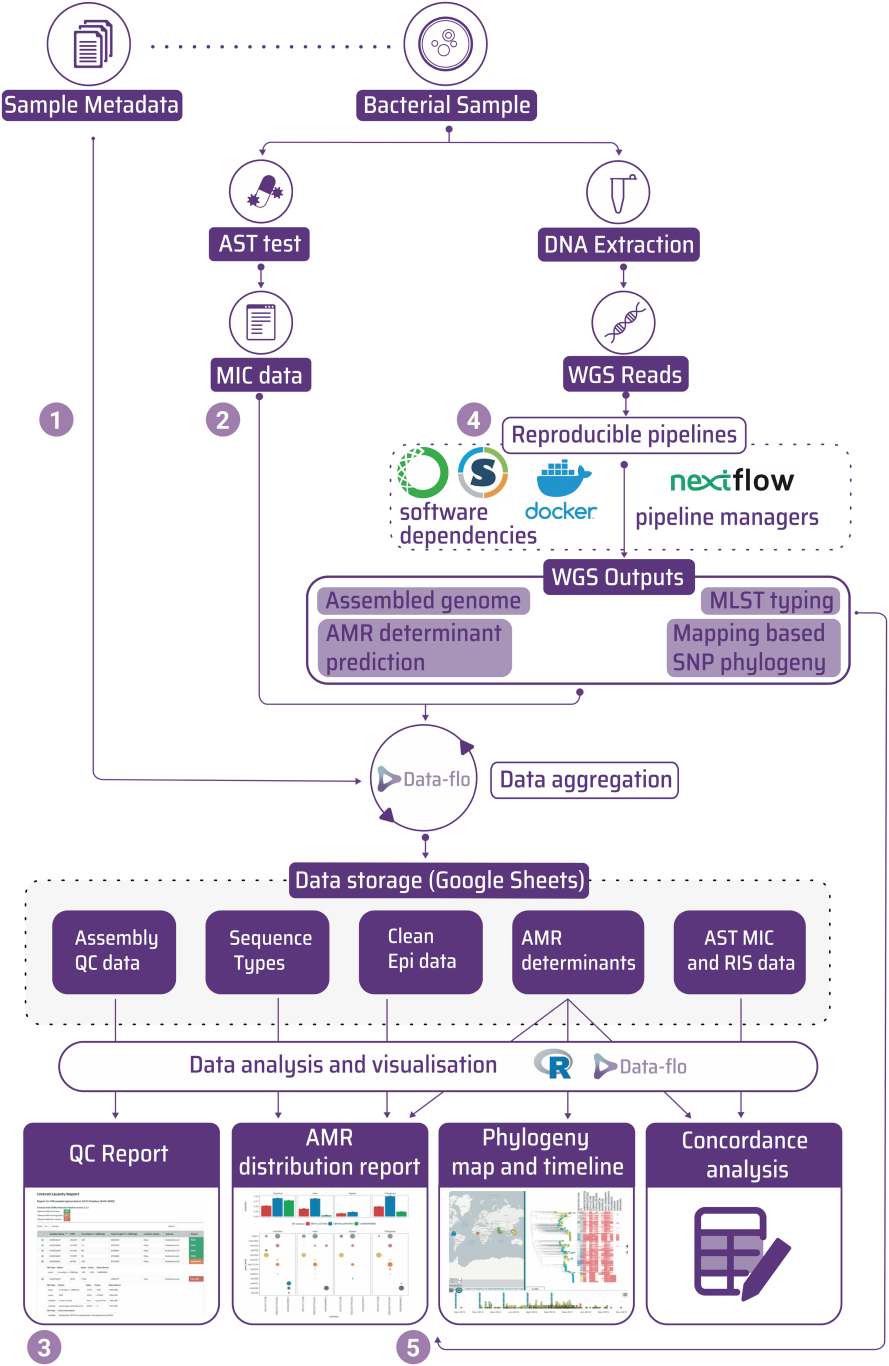
Diagram showing the flow of data from sample receipt to final outputs and highlighting the solutions used for each step. The numbers refer to the same data bottlenecks described in Figure 1. The diagram starts when each bacterial sample is submitted accompanied by associated metadata. The sample is processed by traditional phenotypic antimicrobial sensitivity testing to produce minimum inhibitory concentration data. In parallel, genomic DNA from the sample is extracted and sequenced and whole genome sequencing data are processed through reproducible bioinformatics pipelines to produce multiple outputs such as multilocus sequence type, antimicrobial resistance determinant prediction, and single-nucleotide polymorphism–based phylogenies. These data are aggregated using Data-flo and stored in Google Sheets where they can be combined and manipulated using downstream processes such as R scripts or Data-flo pipeline to make final visualizations or reports. Abbreviations: AMR, antimicrobial resistance; AST, antimicrobial sensitivity testing; MIC, minimum inhibitory concentration; MLST, multilocus sequence typing; QC, quality control; RIS, Resistant, Intermediate, Susceptible; SNP, single-nucleotide polymorphism; WGS, whole genome sequencing.

The GHRU project has sought to address the challenges described in the previous section. We present these as examples of how bottlenecks can be overcome, rather than promoting them as the only or best methods (Figure 2). In addition, the 4 units that have implemented a WGS AMR surveillance pathway present their experiences, unique circumstances, and implementation journeys.

### Data Collection and Integration

Although there are multiple data files and many formats describing metadata, it is usually possible to describe the steps to turn them into consistent formats as a series of defined transformations. There are many alternative data science software solutions for this. We chose to use Data-flo software (https://data-flo.io/), which builds transformation dataflows by combining ready-to-use data adaptors. This technique is very similar to command-line approaches, such as R, or the Python pandas library, but allows users to construct and debug the data flow in a visual way [15, 16]. This enables data science specialists to construct a complex data parsing pipeline and share it with non-bioinformatics staff, such as those responsible for sample receipt, who are then able to run Data-flo without command-line experience. Within the project, we used Data-flo for several purposes: cleaning and transforming sample metadata data before combining with AST outputs (Figures 1 and 2, bottleneck ①; Supplementary Figure 1); converting different VITEK outputs into a single format that could be used as the input source for the WHONET AMR software (Figures 1 and 2, bottleneck ②) [17]; transforming bioinformatics outputs into a readable tabular format before uploading to a Google Sheet for ongoing storage of the results; and reading data from multiple sheets in different Google Sheets and joining them, in order to produce an aggregated table of epidemiological, laboratory, and bioinformatics results.

For example, a downstream Data-flo workflow was constructed to combine epidemiological metadata, AST data, and genomics outputs into the text format required as the source for visualization in Microreact (Figures 1 and 2, bottleneck ⑤) [18]. A suite of programmatic functions was compiled within the ghruR package [19]). However, an important difference is that this did not require a relational database management system to be installed, backed up, and maintained. This would have represented a barrier to sustainability of the data storage solution.

### Sequence Analysis

The analytical procedures to transform raw sequence into meaningful results are complex, often involving multiple steps and software. In order to efficiently parallelize this, so that multiple samples can be processed simultaneously on multicore workstations or in high-performance computing clusters, the use of workflow managers is recommended [20]. Installation of software with specific versions that may differ in different workflows can be one of the main hindrances to running bioinformatics analyses, especially as in many settings a UNIX system administrator with the necessary skills is not available. The use of containerization technology to bundle together specific software versions in a sandboxed environment is the most common solution for this [20–22]. For the GHRU project we took both these approaches, using workflows written for the Nextflow workflow manager, and Docker or Singularity containers to analyze the raw sequence data and produce de novo assemblies, AMR predictions, the 7-locus multilocus sequence type, and mapping-based single-nucleotide polymorphism phylogenies (Figures 1 and 2, bottleneck ④; Supplementary Table 1) [23–25]. To further simplify the software build process, the containers were built using the Conda package management software [26, 27]. This has allowed a total of 5979 genomes from species listed by the WHO as requiring priority research for AMR [28] to be processed across the GHRU partners in Colombia, India, Nigeria, and the Philippines (Supplementary Figure 2), and in parallel at the Wellcome Sanger Institute to confirm reproducibility.

The computational setup required for this was minimal. In each partner center, samples were processed on either high-specification laptops, or a workstation only requiring the installation of Ubuntu Linux, Java RE, Nextflow, and Docker, with either a simple command-line recipe or automated with ansible [29]. An example of a workstation specification is included in Supplementary Table 2. At Wellcome Sanger Institute, Docker images were converted to Singularity images and run on the high-performance computing Load Sharing Facility (LSF) cluster for a combined total of <24 hours. Analytical outputs were compared and shown to be identical, demonstrating the reproducible nature of the pipelines. A critical aspect to automate the processing of samples for WGS is a simple quality control procedure whereby samples that meet the appropriate studies can be triaged. We developed a Python package named Qualifyr to allow sorting of sample outputs into a red/amber/green status based on quality metrics derived from the fastqc read quality assessment package, confindr, and quast [30–33]. This produces a web-based graphical report as well as a text version for computational parsing, so samples that should be taken for further analysis can be easily assessed (Figure 1 and 2, bottleneck ③; Supplementary Figure 3).

### Training

Bridging the knowledge gap is perhaps the most important requirement for effective implementation of WGS. It is important to consider trainee requirements. When processing pathogen WGS data for public health, there are 2 broad use cases: routine interpretation of the data for epidemiological investigation and intervention, and basic research to extrapolate the routine results and explore hypotheses. The first use case requires training to be able to run analytical pipelines and interpret the results, whereas the second use case requires additional training to be able to run more in-depth analyses. In this project, a tiered training program was developed. An example of a training stream for genome assembly is shown in Table 1 [29].

**Table 1.** Genome Assembly Training Stream

Tier Outcome	Tier Title	User Proficiency	Notes
Understand the principles and be able to perform hands-on analysis using web tools	Genome assembly tutorial: principles and web-based analysis	Genomic scientist	Use the Galaxy web platform to run examples samples and assess output
Be able to implement analysis using command line	Genome assembly tutorial: command-line analysis	Command-line user	Run assembly with the command-line tools underpinning the Galaxy interface. The same parameters are employed so that the connection between running the assembly via the website and on the command line is apparent.
Be able to run reproducible high-throughput analysis and interpret the results	Genome assembly tutorial: reproducible batch processing	Command-line user	An in-depth knowledge of the command line is not required for this training.

In this example, a public health bioinformatician would likely take the first and last tiers if they are going to run pipelines. An academic research bioinformatician would take the second tier in addition, in order to understand the commands used so they could make edits to the pipeline if required.

The training was enhanced logistically by delivering some of the command-line tutorial through shared terminal sessions (eg, with Tmux), allowing interactive training. This was supported by using a forum style chat application to allow trainees to talk and assist one another across different time zones [34]. A relatively stable internet connection is therefore a crucial requirement for collaboration in this kind of project. Once training was completed, a series of versioned standard operating procedures were designed to help the project users follow uniform procedures when generating results [35]. An important part of the training aimed to equip local bioinformaticians to train others [10]. Use of these training materials combined with application of the pedagogical techniques taught in the train-the-trainer course has allowed the GHRU units to train public health scientists and researchers outside of their own teams to run the same reproducible analyses [36].

## IMPLEMENTATION VIGNETTES

**Figure 3.**
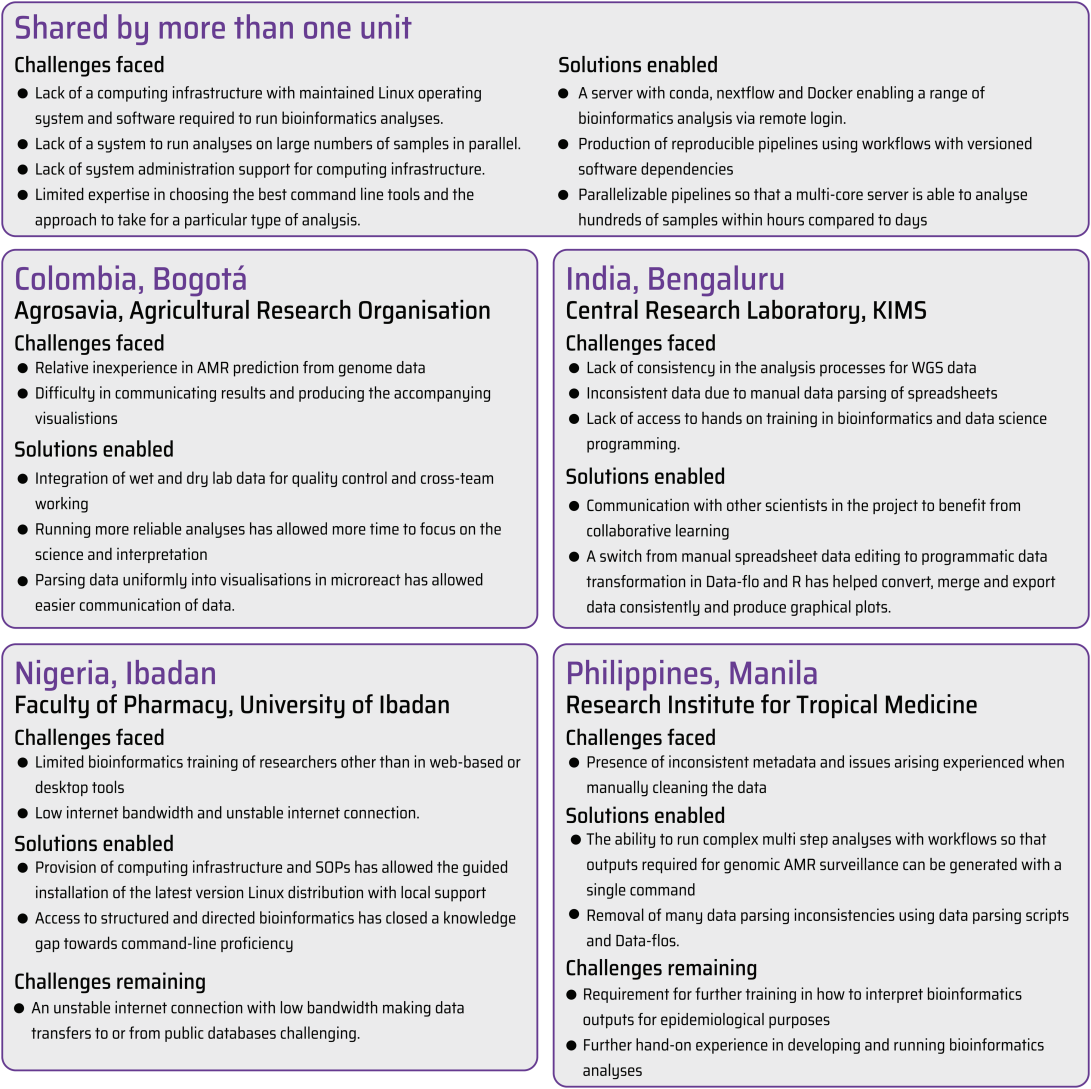
Implementation vignettes. Abbreviations: AMR, antimicrobial resistance; KIMS, Kempegowda Institute of Medical Sciences; SOP, standard operating procedure; WGS, whole genome sequencing.

One of the GHRU’s primary objectives is to build sustainable capacity in AMR laboratories. The goal is for each laboratory to be self-sufficient in the processes of laboratory sequencing and WGS analysis of samples collected for the purposes of AMR surveillance. Each laboratory has a unique set of local circumstances and objectives. These are described in Figure 3, in conjunction with the major milestones achieved.

## Discussion

There are many significant potential hurdles to overcome when implementing genomics for routine pathogen surveillance. Through the use of standardized bioinformatics and a structured training program, 4 institutes in different countries and at different starting points along the genomics journey have been able to process hundreds of pathogen genomes from many clinically significant species. Further developments are still required to ensure sustainable and reliable analysis and interpretation of genome data for AMR surveillance. To check competency, running regular internal quality assessments and taking part in an external quality assessment (EQA) scheme will be essential. The GHRU has designed and implemented a pilot EQA that is currently in progress in the low- and middle-income country partner units. It has been reported that multiple bioinformatics processes for prediction of AMR are often discordant with each other and with phenotypic result, emphasizing the need for quality control standards, which an accredited EQA will partially address [37]. This also highlights the need for standardization in AMR databases. Currently, there are several widely used databases containing catalogues of AMR determinants. The incompatibility between these databases in terms of the nomenclature, use of different reference sequences for allelic variants, and nonstandardized assignation of determinants to resistance to a specific antimicrobial is one of the remaining challenges facing those working in the field of genomic AMR surveillance. The hAMRonization software aims to rationalize the outputs from different AMR software, but a greater need remains the harmonization of the nomenclature and assignations of AMR determinants [38, 39]. This will be crucial for constructing a globally viewable database that stores genome-based AMR data, which is one of the future aims of the WHO Global Antimicrobial Resistance Surveillance System (GLASS) [40].

Future directions for the GHRU project include implementing cloud-based pipelines [41]. The advantages include no requirement for physical workstation procurement, installation, or maintenance, resulting in overall cost savings. However, slow and intermittent internet bandwidth may prove an insurmountable barrier in some locations. The workflow manager Nextflow that was used in the project already has an interface to launch pipelines in cloud computing environments [42]. Training will also be improved using online notebooks such as Google Colaboratory and Kaggle, so that trainees can take advantage of command-line training without requiring a Linux machine and with interactive examples [43, 44]. Additional training in interpretation of results as well as running analyses will be implemented, because the genetic factors contributing to phenotypic AMR are complex. Furthermore, our understanding of the genetics of AMR is constantly changing, and therefore training to build skills that allow the assessment and incorporation of new software into pipelines will be required to ensure sustainability. Together, these iterative improvements will further lower the barriers for bioinformatics implementation for WGS-based surveillance.

## Conclusions

There are several alternate routes to implementing pathogen genome sequencing for surveillance of AMR, each of which has its own strengths. Here we have set out principles for overcoming some of the challenges. The fundamental process is running reproducible analytical pipelines so that consistent results are obtained from multiple centers, as measured by the text/digital output from the pipelines, prior to interpretation. Just as important are the processes to clean incoming data pertaining to the samples, and the downstream processes to amalgamate these with the genomic outputs. We chose to use online spreadsheets as a proxy for database tables and programmatic means to fetch, join, and filter these to produce aggregated data that could be tabulated or visualized. Training was crucial for the implementation and allowed bioinformatics scientists to both run standardized pipelines and perform more specialized, species-directed analyses. For those wanting to run consistent pipelines, online platforms offer capacity without requiring more in-depth bioinformatics expertise. Pathogenwatch offers a web-based platform for AMR analysis and phylogeny generation of *Campylobacter*, *Klebsiella*, *Neisseria gonorrhoeae*, *Staphylococcus aureus*, and *Salmonella* Typhi [13, 14]. The Center for Genomic Epidemiology website offers services for phylogenetic tree building and AMR prediction [45]. However, when using these services it is important to realize that the underlying analyses may change and recording the exact version of the analysis software becomes more challenging.

In the long term, the WHO GLASS system will accept genomic-based AMR prediction and supporting information [40]. Several factors will be important for its success: a mechanism to check the quality of incoming data; a standardized data format; and a minimal data specification. The implementation described here will help inform these factors.

## Supplementary Data

Supplementary materials are available at *Clinical Infectious Diseases* online. Consisting of data provided by the authors to benefit the reader, the posted materials are not copyedited and are the sole responsibility of the authors, so questions or comments should be addressed to the corresponding author.

ciab785_suppl_Supplementary_Material

## Notes

***Acknowledgments.*** Members of the NIHR Global Health Research Unit on Genomic Surveillance of Antimicrobial Resistance: Harry Harste, Mihir Kekre, Dawn Muddyman, Ben Taylor, Nicole Wheeler, and Sophia David (Centre for Genomic Pathogen Surveillance, Big Data Institute, University of Oxford, Old Road Campus, Oxford, United Kingdom [UK] and Wellcome Genome Campus, Hinxton, UK); Alejandra Arevalo, Maria Fernanda Valencia, and Erik C. D. Osma Castro (Colombian Integrated Program for Antimicrobial Resistance Surveillance—Coipars, Centro de Investigación Tibaitatá, Corporación Colombiana de Investigación Agropecuaria, Tibaitatá–Mosquera, Cundinamarca, Colombia); Geetha Nagaraj, Vandana Govindan, Akshata Prabhu, D. Sravani, M. R. Shincy, Steffimole Rose, and Kundur N. Ravishankar (Central Research Laboratory, Kempegowda Institute of Medical Sciences, Bengaluru, India); Anderson O. Oaikhena, Jolaade J. Ajiboye, and Erkison Ewomazino Odih (Department of Pharmaceutical Microbiology, Faculty of Pharmacy, University of Ibadan, Oyo State, Nigeria); Marietta L. Lagrada, Polle Krystle V. Macaranas, Agnettah M. Olorosa, and Elmer M. Herrera (Antimicrobial Resistance Surveillance Reference Laboratory, Research Institute for Tropical Medicine, Muntinlupa, Philippines); Ali Molloy (alimolloy.com); John Stelling (Brigham and Women’s Hospital, Boston, Massachusetts); and Carolin Vegvari (Imperial College London, London, United Kingdom). Figures 1 and 2 include icons that were designed based on resources from Flaticon.com.

***Disclaimer.*** The views expressed in this publication are those of the authors and not necessarily those of the National Health Service, the NIHR, or the UK Department of Health.

***Financial support.*** This work was supported by Official Development Assistance (ODA) funding from the National Institute for Health Research [grant number 16_136_111] and the Wellcome Trust grant number 206194 and the World Health Organization (WHO) (to M. L. M. and S. S.). I. N. O. is supported by an African Research Leader Award from the UK Medical Research Council (MRC) and the UK Department for International Development (DFID) under the MRC/DFID Concordat agreement that is also part of the EDCTP2 program supported by the European Union (I. N. O., University of Ibadan: Pathogenic lineages of enteric bacteria in Nigeria, MR/L00464X/1). This research was commissioned by the National Institute for Health Research using Official Development Assistance (ODA) funding. The views expressed in this publication are those of the authors and not necessarily those of the NHS, the National Institute for Health Research or the Department of Health.

***Supplement sponsorship.*** The supplement is sponsored by the UK National Institute for Health Research Global Health Research Unit on Genomic Surveillance of AMR.

***Potential conflicts of interest.*** I. N. O. is a member of the Bill & Melinda Gates Foundation Surveillance Advisors Group (from 2019); received grants and nonfinancial support from International Vaccine Institute (SETA Award to University of Ibadan); and reports grants from the NIHR Global Health Research Unit on Genomic Surveillance of Antimicrobial Resistance Consortium, during the conduct of the study. K. L. R. reports a travel support grant from the NIHR, outside the submitted work. M. L. M. and S. S. report payments made to their institution by the WHO, outside the submitted work. M. A. reports support from Wellcome Connecting Science, during the conduct of the study. V. S. reports receiving a travel support grant, paid to their institution, from the NIHR, outside the submitted work.

All other authors report no potential conflicts of interest.

## References

[1] Armstrong GL, MacCannell DR, Taylor J, et al. Pathogen genomics in public health. N Engl J Med 2019; 381:2569–80. 31881145 10.1056/NEJMsr1813907 PMC7008580

[2] Hendriksen RS, Bortolaia V, Tate H, Tyson GH, Aarestrup FM, McDermott PF. Using genomics to track global antimicrobial resistance. Front Public Health 2019; 7:242. 31552211 10.3389/fpubh.2019.00242 PMC6737581

[3] World Health Organization. GLASS whole-genome sequencing for surveillance of antimicrobial resistance. 2020. Available at: https://www.who.int/publications/i/item/9789240011007. Accessed 29 September 2021.

[4] Ellington MJ, Ekelund O, Aarestrup FM, et al. The role of whole genome sequencing in antimicrobial susceptibility testing of bacteria: report from the EUCAST subcommittee. Clin Microbiol Infect 2017; 23:2–22. 27890457 10.1016/j.cmi.2016.11.012

[5] Su M, Satola SW, Read TD. Genome-based prediction of bacterial antibiotic resistance. J Clin Microbiol 2019; 57:e01405-18. Available at: https://jcm.asm.org/content/57/3/e01405-18. Accessed 30 September 2020. 30381421 10.1128/JCM.01405-18 PMC6425178

[6] Argimón S, Masim MAL, Gayeta JM, et al. Integrating whole-genome sequencing within the national antimicrobial resistance surveillance program in the Philippines. Nat Commun 2020; 11:2719. 32483195 10.1038/s41467-020-16322-5 PMC7264328

[7] Rossen JWA, Friedrich AW, Moran-Gilad J; ESCMID Study Group for Genomic and Molecular Diagnostics (ESGMD). Practical issues in implementing whole-genome-sequencing in routine diagnostic microbiology. Clin Microbiol Infect 2018; 24:355–60. 29117578 10.1016/j.cmi.2017.11.001

[8] Kekre M, Arevalo SA, Valencia MF, et al. Integrating scalable genome sequencing into microbiology laboratories for routine AMR surveillance. Clin Infect Dis 2021; 73:XX–XXX. 10.1093/cid/ciab796 PMC8634525 34850836

[9] Fricke WF, Rasko DA. Bacterial genome sequencing in the clinic: bioinformatic challenges and solutions. Nat Rev Genet 2014; 15:49–55. 24281148 10.1038/nrg3624

[10] Abrudan M, Matimba A, Nikolic D, et al. Train-the-trainer as an effective approach to building global networks of experts in genomic surveillance of AMR. Clin Infect Dis 2021; 73:XX–XXX. 10.1093/cid/ciab770 PMC8634536 34850831

[11] Afgan E, Baker D, Batut B, et al. The Galaxy platform for accessible, reproducible and collaborative biomedical analyses: 2018 update. Nucleic Acids Res 2018; 46:W537–44. 29790989 10.1093/nar/gky379 PMC6030816

[12] Thomsen MCF, Ahrenfeldt J, Cisneros JLB, et al. A bacterial analysis platform: an integrated system for analysing bacterial whole genome sequencing data for clinical diagnostics and surveillance. PLoS One 2016; 11:e015778. 10.1371/journal.pone.0157718 PMC4915688 27327771

[13] Argimón S, Yeats CA, Goater RJ, et al. A global resource for genomic predictions of antimicrobial resistance and surveillance of *Salmonella* Typhi at Pathogenwatch. Nat Commun 2021; 12:2879. 34001879 10.1038/s41467-021-23091-2 PMC8128892

[14] Sánchez-Busó L, Yeats CA, Taylor B, et al. A community-driven resource for genomic surveillance of Neisseria gonorrhoeae at Pathogenwatch. Genome Med 2021; 13:61. 10.1186/s13073-021-00858-2 PMC8054416 33875000

[15] R Core Team. R: A Language and Environment for Statistical Computing. Vienna, Austria: R Foundation for Statistical Computing, 2020.

[16] McKinney W . Data structures for statistical computing in python. Proc 9th Python Sci Conf 2010; 445: 56–61.

[17] Stelling JM, O’Brien TF. Surveillance of antimicrobial resistance: the WHONET program. Clin Infect Dis 1997; 24(Suppl 1):S157–68. 8994799 10.1093/clinids/24.supplement_1.s157

[18] Argimón S, Abudahab K, Goater RJE, et al. Microreact: visualizing and sharing data for genomic epidemiology and phylogeography. Microb Genom 2016; 2:e000093. 28348833 10.1099/mgen.0.000093 PMC5320705

[19] Underwood A . ghruR. Available at: https://gitlab.com/cgps/ghru/ghrur. Accessed 6 November 2020.

[20] Strozzi F, Janssen R, Wurmus R, et al. Scalable workflows and reproducible data analysis for genomics. In: Anisimova M, ed. Evolutionary Genomics: Statistical and Computational Methods. New York: Springer, 2019:723–45. 10.1007/978-1-4939-9074-0_24 PMC7613310 31278683

[21] Ewels PA, Peltzer A, Fillinger S, et al. The nf-core framework for community-curated bioinformatics pipelines. Nat Biotechnol 2020; 38:276–8. 32055031 10.1038/s41587-020-0439-x

[22] State Public Health Bioinformatics Group. Welcome to StaPH-B. Available at: https://staph-b.github.io/. Accessed 26 November 2020.

[23] Di Tommaso P, Chatzou M, Floden EW, Barja PP, Palumbo E, Notredame C. Nextflow enables reproducible computational workflows. Nat Biotechnol 2017; 35:316–9. 28398311 10.1038/nbt.3820

[24] Merkel D . Docker: lightweight Linux containers for consistent development and deployment. Linux J 2014; 2014:239.

[25] Kurtzer GM, Sochat V, Bauer MW. Singularity: scientific containers for mobility of compute. PLoS One 2017; 12:e0177459. 28494014 10.1371/journal.pone.0177459 PMC5426675

[26] Anaconda. The world’s most popular data science platform. Available at: https://www.anaconda.com/. Accessed 7 October 2020.

[27] GitLab. SNP phylogeny Conda environment. Available at: https://gitlab.com/cgps/ghru/pipelines/snp_phylogeny/-/blob/master/environment.yml. Accessed 7 October 2020.

[28] World Health Organization. WHO publishes list of bacteria for which new antibiotics are urgently needed. Available at: https://www.who.int/news/item/27-02-2017-who-publishes-list-of-bacteria-for-which-new-antibiotics-are-urgently-needed. Accessed 15 December 2020.

[29] Center for Genomic Pathogen Surveillance. CGPS protocols. Available at: https://www.pathogensurveillance.net/resources/protocols/. Accessed 15 December 2020.

[30] GitLab. Qualifyr. Available at: https://gitlab.com/cgps/qualifyr. Accessed 7 October 2020.

[31] Babraham Bioinfomatics. FastQC: a quality control tool for high throughput sequence data. Available at: https://www.bioinformatics.babraham.ac.uk/projects/fastqc/. Accessed 7 October 2020.

[32] Low AJ, Koziol AG, Manninger PA, Blais B, Carrillo CD. ConFindr: rapid detection of intraspecies and cross-species contamination in bacterial whole-genome sequence data. PeerJ 2019; 7:e6995. 31183253 10.7717/peerj.6995 PMC6546082

[33] Gurevich A, Saveliev V, Vyahhi N, Tesler G. QUAST: quality assessment tool for genome assemblies. Bioinformatics 2013; 29:1072–5. 23422339 10.1093/bioinformatics/btt086 PMC3624806

[34] Doist. Twist: clear and organized team communication. Available at: https://twist.com/. Accessed 8 March 2021.

[35] National Institute for Health Research. GHRU de novo assembly standard operating procedure. Available at: https://docs.google.com/document/u/2/d/1_CY9U3IOducJnBt0eTm3gARoT-n4VTIUv5X_hoCnQN4/edit?usp=drive_web&ouid=104754426691844314353&usp=embed_facebook. Accessed 8 October 2020.

[36] Center for Genomic Pathogen Surveillance. GHRU protocols. Available at: https://www.pathogensurveillance.net/resources/protocols/. Accessed 26 February 2021.

[37] Doyle RM, O’Sullivan DM, Aller SD, et al. Discordant bioinformatic predictions of antimicrobial resistance from whole-genome sequencing data of bacterial isolates: an inter-laboratory study. Microb Genomics 2020; 6:e000335. 10.1099/mgen.0.000335 PMC7067211 32048983

[38] hAMRonization. Public Health Alliance for Genomic Epidemiology. 2020. Available at: https://github.com/pha4ge/hAMRonization. Accessed 8 October 2020.

[39] Mahfouz N, Ferreira I, Beisken S, von Haeseler A, Posch AE. Large-scale assessment of antimicrobial resistance marker databases for genetic phenotype prediction: a systematic review. J Antimicrob Chemother 2020; 75:3099–108. 32658975 10.1093/jac/dkaa257 PMC7566382

[40] World Health Organization. Global antimicrobial resistance and use surveillance system (GLASS) report. 2020. Available at: http://www.who.int/glass/resources/publications/early-implementation-report-2020/en/. Accessed 8 March 2021.

[41] Navale V, Bourne PE. Cloud computing applications for biomedical science: a perspective. PLoS Comput Biol 2018; 14:e1006144. 29902176 10.1371/journal.pcbi.1006144 PMC6002019

[42] Nextflow Tower. Get started. Available at: https://tower.nf/. Accessed 8 October 2020.

[43] Google Colaboratory. What is colaboratory? Available at: https://colab.research.google.com/notebooks/intro.ipynb. Accessed 8 October 2020.

[44] Kaggle. Available at: https://www.kaggle.com/. Accessed 8 October 2020.

[45] Center for Genomic Epidemiology. CGE server. Available at: https://cge.cbs.dtu.dk/services/. Accessed 21 October 2020.

