# Supplementary material for: Overcoming Data Bottlenecks in Genomic Pathogen Surveillance
Source: Clin Infect Dis. 2021 Nov 25;73(Suppl 4):S267–74. doi: 10.1093/cid/ciab785 (PMC8634317; doi:10.1093/cid/ciab785)
Supplement: ciab785_suppl_Supplementary_Material [file ciab785_suppl_supplementary_material.docx]

**Supplementary Information**

**Overcoming Data Bottlenecks in Genomic Pathogen Surveillance**

Ayorinde O. Afolayan^4^, Johan F. Bernal^5^, June Gayeta^2^, Melissa Masim^2,^ Varun Shamanna^3^, Monica Abrudan^1^, Khalil Au-Dahab^1^, Silvia Argimon^1^, Celia Carlos^2^, Sonia Sia^2^, Ravi Kumar^3^, Iruka N. Okeke^4^, Pilar Donado-Godoy^5^, and David M. Aanensen^1^, Anthony Underwood^1^ and the NIHR Global Health Research Unit on Genomic Surveillance of Antimicrobial Resistance ^a^

**Affiliations**

1. Centre for Genomic Pathogen Surveillance, Big Data Institute, University of Oxford, Old Road Campus, Oxford, United Kingdom and Wellcome Genome Campus, Hinxton, UK.

2. Antimicrobial Resistance Surveillance Reference Laboratory, Research Institute for Tropical Medicine, Muntinlupa, the Philippines.

3. Central Research Laboratory, Kempegowda Institute of Medical Sciences, Bengaluru, India.

4. Global Health Research Unit on Genomic Surveillance of Antimicrobial Resistance, Department of Pharmaceutical Microbiology, Faculty of Pharmacy, University of Ibadan, Oyo State, Nigeria.

5. Colombian Integrated Program for Antimicrobial Resistance Surveillance – Coipars, CI Tibaitatá, Corporación Colombiana de Investigación Agropecuaria (AGROSAVIA), Tibaitatá – Mosquera, Cundinamarca, Colombia.

^a^ Members of the NIHR Global Health Research Unit on Genomic Surveillance of Antimicrobial Resistance are listed in the Acknowledgments.

**BIOINFORMATICS METHODS**

All genome sequence data were processed using versioned Nextflow [1] workflows and associated Docker [2] containers covering the foundational analyses of de novo assembly, mapping-based SNP phylogeny, MLST assignation and AMR determinant detection (Supplementary Table 1). The exact steps performed can be derived from examination of the pipeline code, but each workflow will be described in brief.

De novo assembly: reads trimming and adapter removal using trimmomatic (0.38) [3], read correction using lighter (1.1.1) [4], downsampling to 100x coverage using seqtk (1.3) [5], read merging using flash (1.2.11) [6], assembly using SPAdes (3.12.0) [7]. Quality control was performed using fastqc (0.11.8) [8], multiqc (1.7) [9] and qualifyr (1.4.4) [10]. Species identification was carried out by bactinspector (0.1.3) [11] and contamination checked using Confindr (0.7.2) [12].

Mapping based phylogeny: Reads were trimmed as described for de novo assembly and mapped to a reference with bwa mem (0.7.17) [13], variants called and filtered using bcf tools (1.9) [14] and the filtering conditions for a low quality position being '%QUAL<25 || FORMAT/DP<10 || MAX(FORMAT/ADF)<2 || MAX(FORMAT/ADR)<2 || MAX(FORMAT/AD)/SUM(FORMAT/DP)<0.9 || MQ<30 || MQ0F>0.1'. A pseudoalignment where each sample has a base relative to the reference sequence. Missing bases and low-quality bases are encoded using the - and N characters. The alignment was used to generate a maximum likelihood tree using iqtree (1.6.8) [15, 16] and ultrafast bootstraps (parameters -m GTR+G -alrt 1000 -bb 1000).

AMR determinant detection: The ARIBA software (2.14.4) [17] was used to detect acquired genes using the NCBI database [18] (downloaded 2019-10-30) and the pointfinder database (downloaded 2020-12-11) adapted for Ariba [19].

MLST detection: The ARIBA software (2.14.4) [17] was used to determine the 7-locus MLST type using the profile and alleles found in the pubmlst database [20, 21] (downloaded 2020-12-20).

1. Di Tommaso P, Chatzou M, Floden EW, Barja PP, Palumbo E, Notredame C. Nextflow enables reproducible computational workflows. Nat Biotechnol **2017**; 35:316–319.

2. Merkel, D. Docker: lightweight linux containers for consistent development and deployment. **2014**; 239.

3. Bolger AM, Lohse M, Usadel B. Trimmomatic: a flexible trimmer for Illumina sequence data. Bioinforma Oxf Engl **2014**; 30:2114–2120.

4. Song L, Florea L, Langmead B. Lighter: fast and memory-efficient sequencing error correction without counting. Genome Biol **2014**; 15:509.

5. Li H. lh3/seqtk. 2020. Available at: https://github.com/lh3/seqtk. Accessed June 17, 2021.

6. Magoč T, Salzberg SL. FLASH: fast length adjustment of short reads to improve genome assemblies. Bioinformatics **2011**; 27:2957–2963.

7. Bankevich A, Nurk S, Antipov D, et al. SPAdes: a new genome assembly algorithm and its applications to single-cell sequencing. J Comput Biol J Comput Mol Cell Biol **2012**; 19:455–477.

8. FastQC A Quality Control tool for High Throughput Sequence Data. Available at: https://www.bioinformatics.babraham.ac.uk/projects/fastqc/. Accessed June 17, 2021.

9. Ewels P, Magnusson M, Lundin S, Käller M. MultiQC: summarize analysis results for multiple tools and samples in a single report. Bioinforma Oxf Engl **2016**; 32:3047–3048.

10. Qualifyr. Available at: https://gitlab.com/cgps/qualifyr. Accessed June 17, 2021.

11. BactInspector. Available at: https://gitlab.com/antunderwood/bactinspector. Accessed June 17, 2021.

12. Low AJ, Koziol AG, Manninger PA, Blais B, Carrillo CD. ConFindr: rapid detection of intraspecies and cross-species contamination in bacterial whole-genome sequence data. PeerJ **2019**; 7:e6995.

13. Li H. Aligning sequence reads, clone sequences and assembly contigs with BWA-MEM. ArXiv13033997 Q-Bio 2013; Available at: http://arxiv.org/abs/1303.3997. Accessed June 17, 2021.

14. samtools/bcftools. samtools, 2020. Available at: https://github.com/samtools/bcftools. Accessed June 17, 2021.

15. Nguyen L-T, Schmidt HA, von Haeseler A, Minh BQ. IQ-TREE: A Fast and Effective Stochastic Algorithm for Estimating Maximum-Likelihood Phylogenies. Mol Biol Evol **2015**; 32:268–274.

16. Hoang DT, Chernomor O, von Haeseler A, Minh BQ, Vinh LS. UFBoot2: Improving the Ultrafast Bootstrap Approximation. Mol Biol Evol **2018**; 35:518–522.

17. Hunt M, Mather AE, Sánchez-Busó L, et al. ARIBA: rapid antimicrobial resistance genotyping directly from sequencing reads. Microb Genomics **2017**; 3:e000131.

18. Bacterial Antimicrobial Resistance Reference Gene. Available at: https://www.ncbi.nlm.nih.gov/bioproject/PRJNA313047. Accessed June 17, 2021.

19. ariba_amr_databases. Available at: https://gitlab.com/cgps/ghru/pipelines/data_sources/ariba_amr_databases. Accessed June 17, 2021.

20. Jolley KA, Chan M-S, Maiden MCJ. mlstdbNet - distributed multi-locus sequence typing (MLST) databases. BMC Bioinformatics **2004**; 5:86.

21. Jolley KA, Maiden MCJ. BIGSdb: Scalable analysis of bacterial genome variation at the population level. BMC Bioinformatics **2010**; 11:595.

**Supplementary Tables**

**Supplementary Table 1.** Nextflow workflows.

| **Workflow name** | **Workflow link** | **Docker hub Container(s) used** | **Version at publication** |
| --- | --- | --- | --- |
| *De novo* assembly | https://gitlab.com/cgps/ghru/pipelines/assembly | bioinformant/ghru-assembly:version OR registry.gitlab.com/cgps/ghru/pipelines/assembly:version | 1.5.5 |
| Mapping based SNP phylogeny | https://gitlab.com/cgps/ghru/pipelines/snp_phylogeny | bioinformant/ghru-snp-phylogeny:version  OR registry.gitlab.com/cgps/ghru/pipelines/snp_phylogeny:version | 1.2.2 |
| AMR prediction | https://gitlab.com/cgps/ghru/pipelines/dsl2/pipelines/amr_prediction | bioinformant/ghru-amr-prediction:version OR registry.gitlab.com/cgps/ghru/pipelines/dsl2/pipelines/amr_prediction | 1.0 |
| MLST | https://gitlab.com/cgps/ghru/pipelines/dsl2/pipelines/mlst | bioinformant/ghru-mlst:version  OR registry.gitlab.com/cgps/ghru/pipelines/dsl2/pipelines/mlst:version | 1.0 |

Supplementary Table 2. Example specification of workstation for running analysis of multiple pathogen samples.

| Workstation component | Specification |
| --- | --- |
| Chassis | Under desk or rack configuration with space for 18 drives |
| CPU | 2 x 12 Core CPU |
| RAM | 192 GB (8GB/core) |
| Operating system drive | 2 x 256GB SSD (RAID 1 Mirror configuration) |
| Data drive | 8 x 8Tb HDD (RAID 6 configuration) |
| Spares | 2 x 8Tb HDD in case of disk failure |

**Supplementary Figures**


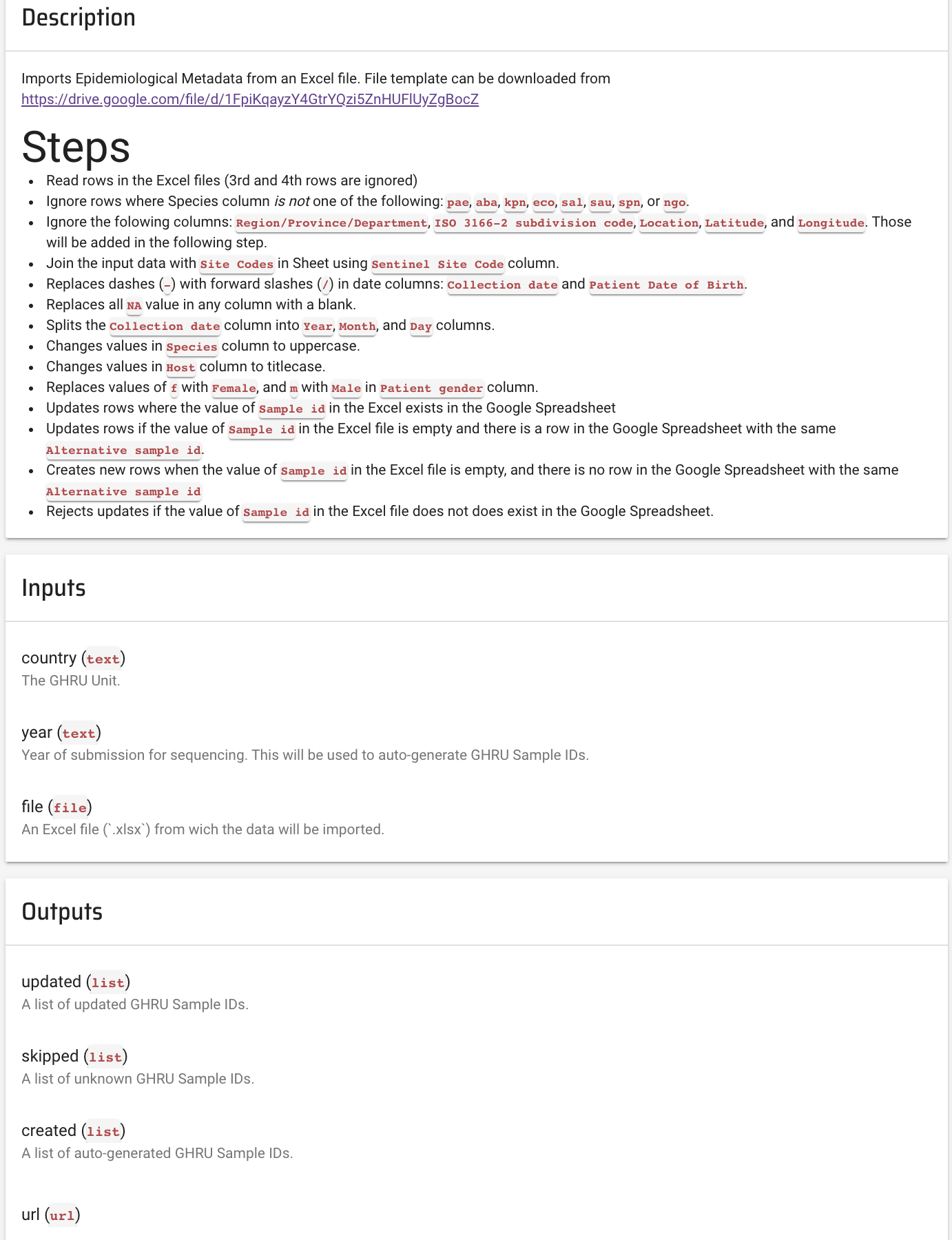


**Supplementary Figure 1.** Description page for the GHRU Data-flo workflow for cleaning and parsing epidemiological metadata.


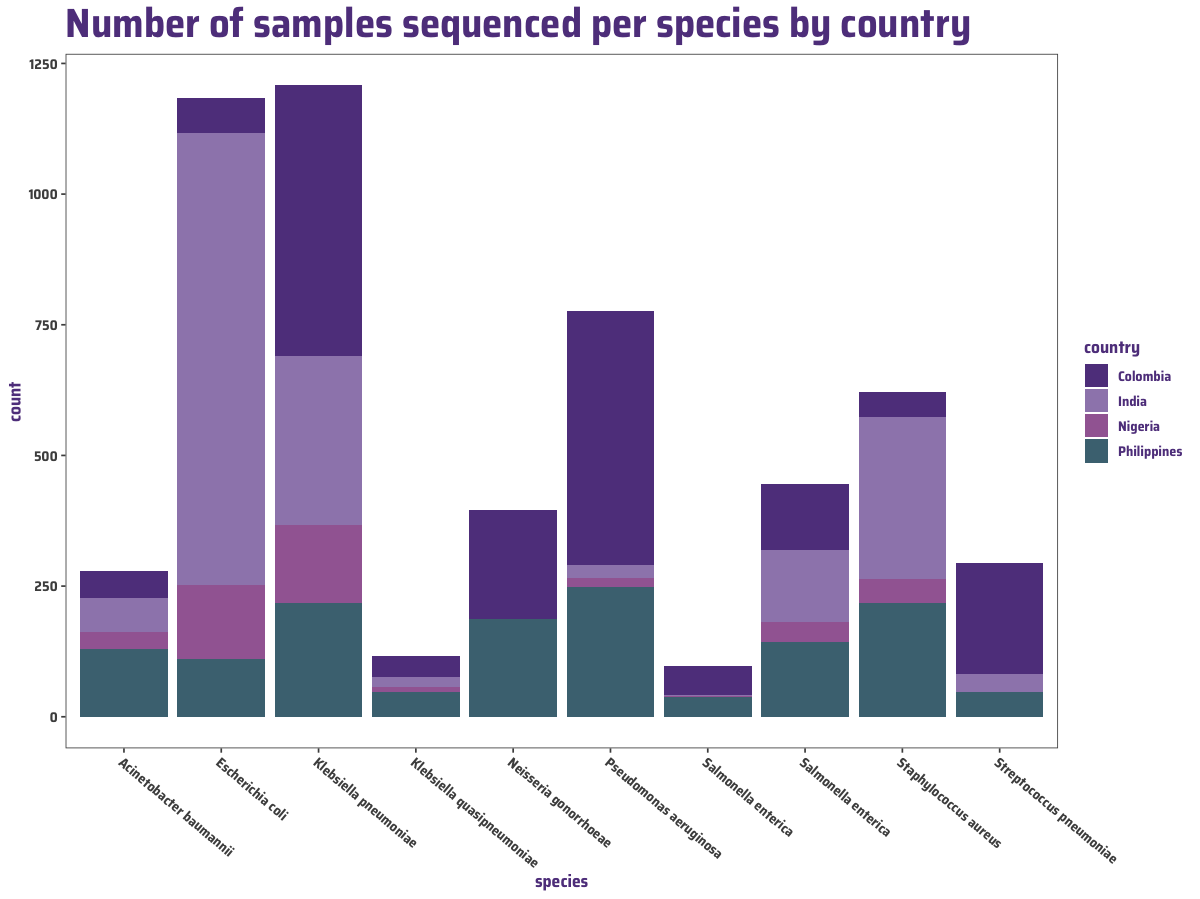


**Supplementary Figure 2** **.** Samples processed through bioinformatics pipelines in each GHRU unit divided by species.


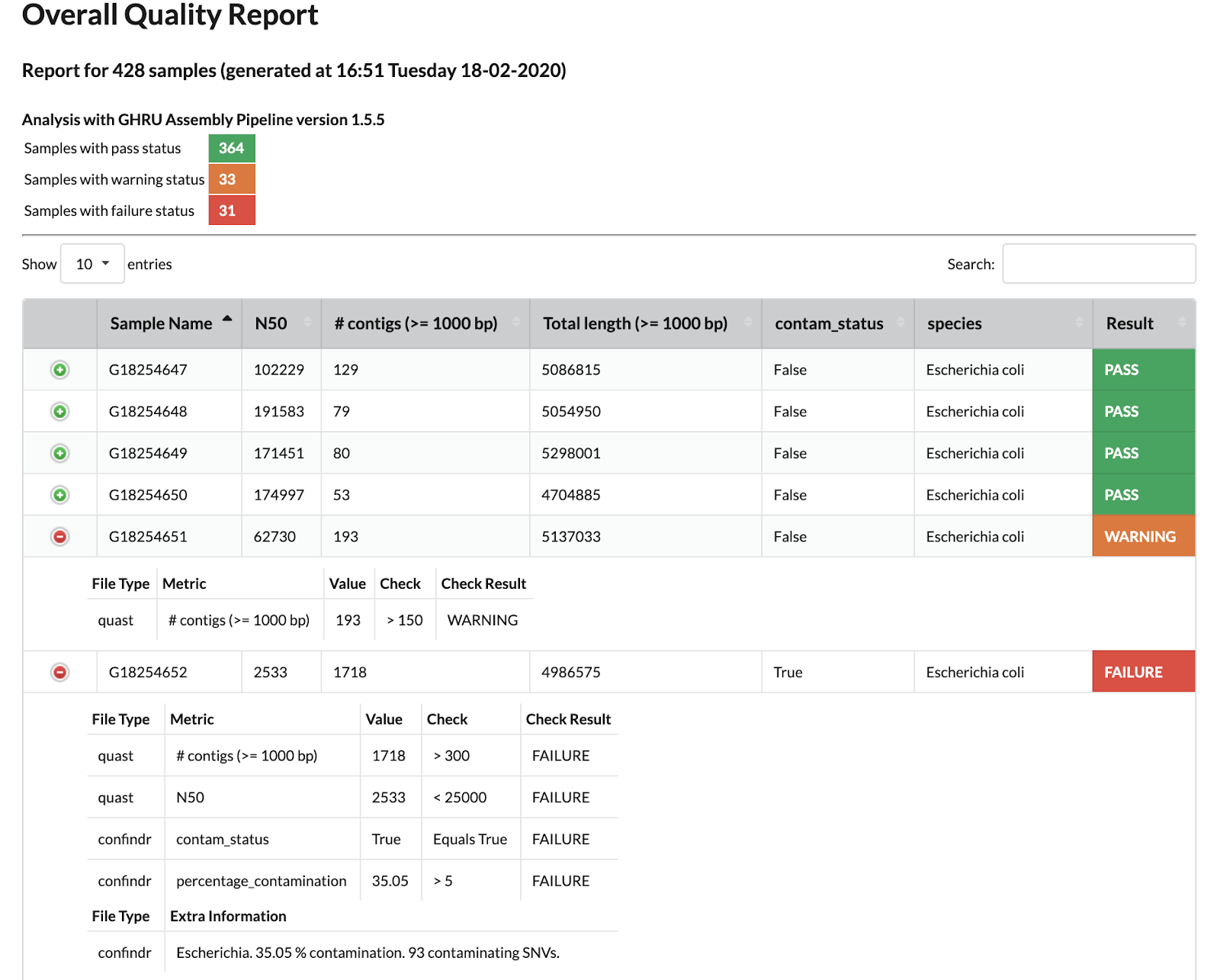


**Supplementary Figure 3** **.** Example output of the web-based graphical report from the Qualifyr software package.
